# The role of mesenchymal stem cells in promoting the transformation of androgen-dependent human prostate cancer cells into androgen-independent manner

**DOI:** 10.1038/srep16993

**Published:** 2016-01-20

**Authors:** Jiwen Cheng, Keqin Yang, Qingyun Zhang, Yang Yu, Qinggui Meng, Ning Mo, Yang Zhou, Xianlin Yi, Chengzhong Ma, Aming Lei, Yan Liu

**Affiliations:** 1 Department of Urology, Affiliated Tumor Hospital of Guangxi Medical University, Nanning, People’s Republic of China; 2 Department of Orthopedics, Guigang city people’s hospital, Guigang, People’s Republic of China; 3 The Fifth Department of Chemotherapy, Affiliated Tumor Hospital of Guangxi Medical University, Nanning, People’s Republic of China; 4 Department of Oncology, Ruikang Hospital Affiliated to Guangxi University of Chinese Medicine, Nanning, People’s Republic of China

## Abstract

Mesenchymal stem cells (MSCs) play an important role in the development of human prostate cancer (PCa). However, the role of MSCs in the transformation of androgen-dependent human PCa cells into androgen-independent manner has been poorly understood. In this study, we investigated the underlying mechanism of MSCs in promoting PCa cells from androgen-dependent into androgen-independent manner. Firstly, we demonstrated that MSCs could affect the transformation of androgen-dependent human PCa cells into androgen-independent manner *in vivo* and *in vitro*. Then we found a substantial expression of TGF-β in MSCs. TGF-β blockade could significantly inhibit the promotive function of MSCs in PCa cells. Besides that, we also demonstrated androgen might inhibit the expression of TGF-β in MSCs. Furthermore, we found that either overexpression of SSEA-4 or the number of SSEA-4 positive MSCs in PCa tissues was associated with a shorter cancer-free survival interval (CFSI) and a worse overall survival (OS). Our results suggest that androgen blockade treatment in clinical PCa therapy may elicit the expression of TGF-β in MSCs, which will result in the transformation of androgen-dependent human PCa cells into androgen-independent manner.

Prostate cancer (PCa) is the most common cancer in men in western countries and is the second leading cause of cancer death in men^1^,^2^, second only to lung cancer. Androgen deprivation therapy is an important method for the clinical treatment of human prostate cancer. However the transformation of androgen-dependent human prostate cancer cells into androgen-independent manner during androgen deprivation therapy is the main obstacle that impacts the treatment. Growing evidence has proved that mesenchymal stem cells (MSCs) may play an important role in the development of prostate cancer^3^,^4^. However, the role of mesenchymal stem cells in the transformation of androgen-dependent human prostate cancer cells into androgen-independent manner is still not fully understood.

MSCs originating from the mesodermal germ layer are a subset of nonhematopoietic stem cells existed in bone marrow^5^,^6^. MSCs were described as an adherent, fibroblast-like population and have the ability to differentiate into multiple lineages such as chondrocytes, osteocytes, adipocytes, myocytes, and astrocytes, which is a potential source of stem cells for cellular and genetic therapy^7^,^8^. MSCs also existed in other tissues, including adipose, umbilical cord, muscle, lung and fetal liver^7^,^9^,^10^,^11^,^12^.

MSCs have a tropism for tumors^13^ and several studies have reported contradictory results about the effect of MSCs on tumor growth. Hall *et al.*^14^ demonstrated that co-culture of ALL cell lines with stromal cells which is overexpressed of VCAM-1 enhanced the survival of leukemic cells in a PI-3 kinase dependent manner compared to the co-culture with stromal cells expressing only endogenous VCAM-1. Djouad *et al.*^15^ showed that MSCs had displayed side effects related to systemic immunosupression favoring tumor growth *in vivo*. On the contrary, MSCs have been reported to be anti-tumorigenic in a mouse model of Kaposi’s sarcoma by inhibiting AKT activity^16^. The stroma tissue is an important part of tumor and MSCs is the progenitor cells of stromal cells. So we suppose that MSCs may play a key role in inducing androgen resistance in human prostate cancer cells. In this study, we used LNCaP prostate cancer cell line to investigate the effect of MSCs on the transformation of androgen-dependent human PCa cells into androgen-independent manner and the potential mechanism.

## Results

### MSCs promote the growth of LNCaP prostate cancer cells in androgen-independent condition *in vivo* and *in vitro*

**Figure 1:**
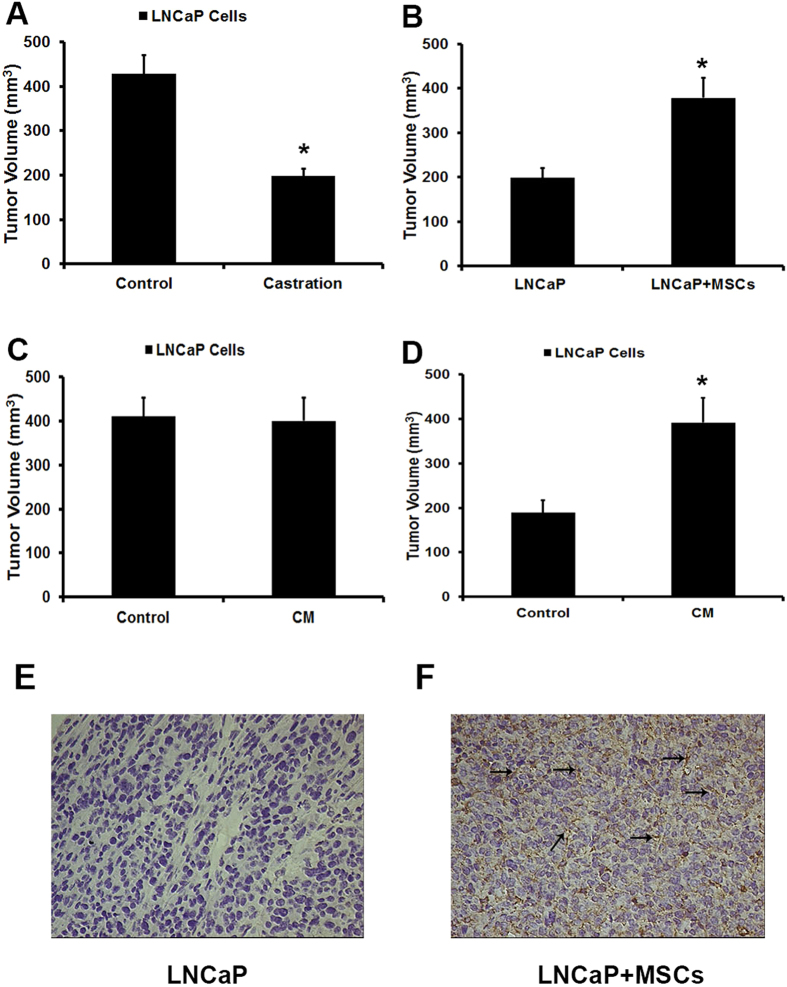
MSCs promote the growth of LNCaP prostate cancer cells in androgen-independent condition *in vivo.* (**A**) LNCaP cells (1 × 10^6^ cells in 200 μL PBS) were subcutaneously administrated in the armpit area of male and castrated nude mice. Mice were examined every day. (**B**) LNCaP cells and MSCs were prepared either as single-cell type suspensions (1 × 10^6^ LNCaP cells in 200 μL PBS) or a mix of cells (1 × 10^6^ LNCaP cells and 2 × 10^5^ MSCs in 200 μL PBS). LNCaP cells (alone or mixed with MSCs) were subcutaneously administrated in the armpit area of castrated nude mice. (**C**) LNCaP cells were pretreated with conditioned medium (CM) collected from MSCs for 5 days and then subcutaneously administrated in the armpit area of male nude mice. (**D**) LNCaP cells were pretreated with conditioned medium (CM) collected from MSCs for 5 days and then subcutaneously administrated in the armpit area of castrated nude mice. On 70 day after implantation, the animals (n = 8/group) were sacrificed and tumor volume was evaluated by measuring the length and width of tumor mass (*P < 0.05). Tumor volume (mm^3^) = 0.52 × width (mm)^2^ × length (mm). (**D,E**) The expression of SSEA-4 in tumors injected with LNCaP cells alone or together with MSCs were stained by immunohistochemistry. The tumors cojected with LNCaP cells and MSCs in castrated male nude mice showed a high expression of SSEA-4 in the tumor stroma (arrowheads). Representative photomicrographs are magnified 400 × .

Firstly, we demonstrated that the development of LNCaP cells in male nude mice was significantly faster than in castrated nude mice. However, when LNCaP cells were coinjected with MSCs in castrated nude mice, the volume of tumor is higher than group injected with LNCaP cells alone (Fig. 1A,B). Besides that, we also collected conditioned medium from MSCs. The conditioned medium was used to pretreat LNCaP cells. Then the pretreated LNCaP cells were implanted not only in male nude mice but also in castrated nude mice. As shown in Fig. 1C, no difference could be observed between the LNCaP cells that pretreated or unpretreated with conditioned medium when they were implanted in male nude mice. However, compared with control groups, a higher development of the tumor in castrated nude mice could be observed in the conditioned medium pretreated group (Fig. 1D).

**Figure 2:**
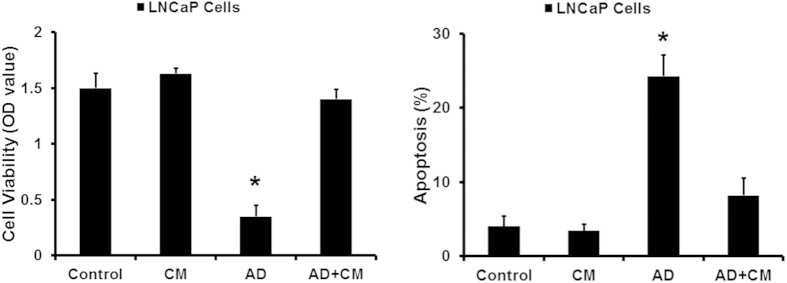
MSCs promote the growth of LNCaP prostate cancer cells in androgen-deprivation condition *in vitro.* CCK8 and PI/Annexin V-FITC assay was used to examined the cell viability and apoptosis of LNCaP cells in an androgen-deprivation (**AD**) condition (*P < 0.05).

Furthermore, the effect of conditioned medium from MSCs was also confirmed by the proliferation and apoptosis of LNCaP cells in androgen deprivation (AD) condition. As shown in Fig. 2, androgen deprivation significantly leaded to the proliferation inhibition and apoptosis enhancement in LNCaP cells. However, when LNCaP cells were treated with conditioned medium from MSCs, their growth showed an androgen-independent manner and the apoptosis decreased.

SSEA-4 has been reported as an effective marker to identify MSCs in tumor tissues^17^,^18^, so we employed SSEA-4 to explore the presence of MSCs in tumors from castrated male nude mice injected with LNCaP cells alone or together with MSCs by immunohistochemistry (IHC). Obviously, compared with the tumors injected with LNCaP cells alone, high expression of SSEA-4 was detected in the stroma of tumors cojected with LNCaP cells and MSCs. MSCs were randomly distributed inside the tumor and the number was relatively high (Fig. 1E,F). These results indicated that MSCs might play a potential role in inducing the growth of LNCaP cells from androgen-dependent into independent manner.

### MSCs promoted the transformation of LNCaP cells via TGF-β

**Figure 3:**
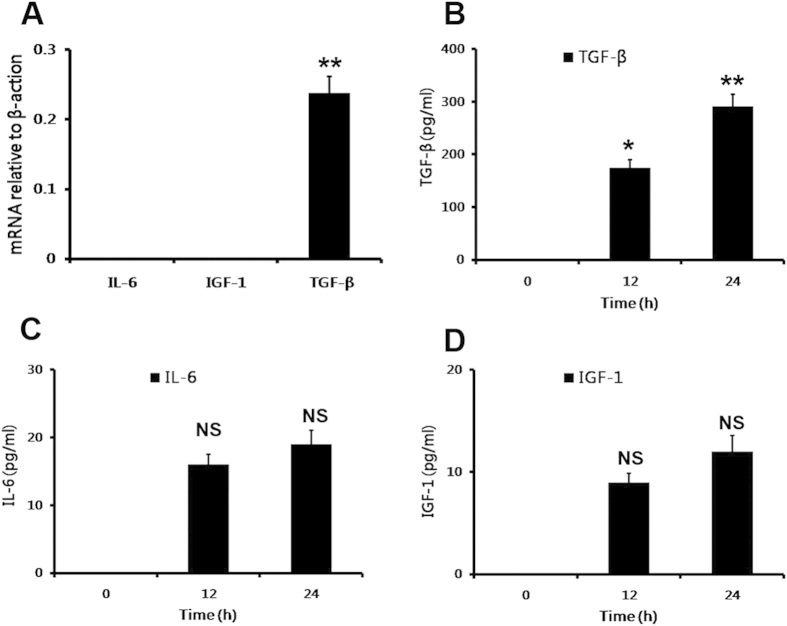
The spontaneous expression of IL-6, TGF-β and IGF-1 in MSCs. (**A**) Realtime PCR was employed to detect the expression of IL-6, TGF-β and IGF-1 in MSCs. (**B**) ELISA assay was also used to examine the expression of IL-6, TGF-β and IGF-1 in the conditioned medium of MSCs collected at 0, 12 and 24 hours (*P < 0.05, **P < 0.01).

Androgen receptor (AR) is a nuclear receptor that has been recognized as a major factor involved in prostate tumor genesis^19^. It also has been reported that IL-6, TGF-β and IGF-1 played an important role in activating AR^20^,^21^. So we examined the expression of IL-6, TGF-β and IGF-1 in MSCs. The results showed that a substantial expression of TGF-β could be detected not only in MSCs but also in the conditioned medium (Fig. 3A,B). However, we did not observe a spontaneously high expression of IL-6 and IGF-1 either in MSCs nor in the conditioned medium of MSCs (Fig. 3A,C,D). The results indicated that the transforming effect of MSCs on LNCaP cells from androgen-dependent into independent manner might be mediated by TGF-β.

**Figure 4:**
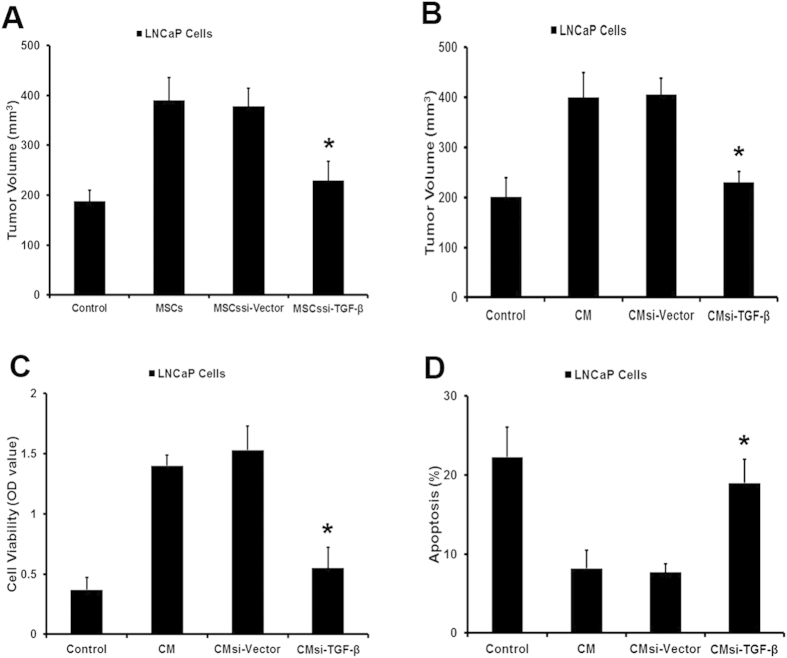
MSCs promoted the transformation of LNCaP cells via TGF-β. We employed siRNA to block the expression of TGF-β in MSCs. (**A**) LNCaP cells and MSCs^si-TGF-β^ were prepared either as single-cell type suspensions (1 × 10^6^ LNCaP cells in 200 μL PBS) or a mix of cells (1 × 10^6^ LNCaP cells and 2 × 10^5^ MSCs^si-TGF-β^ in 200 μL PBS). LNCaP cells (alone or mixed with MSCs) were subcutaneously administrated in the armpit area of castrated nude mice. (**B**) LNCaP cells were pretreated with conditioned medium (CM^si-TGF-β^) collected from MSCs^si-TGF-β^ for 5 days and then subcutaneously administrated in the armpit area of castrated nude mice. On 70 day after implantation, the animals were sacrificed and tumor volume was evaluated by measuring the length and width of tumor mass. (**C,D**) pretreated with CMsi-TGF-β in an androgen-deprivation (**AD**) condition (*P < 0.05).

In order to confirm the function of TGF-β, we used shRNA to inhibit the expression of TGF-β in MSCs. Then function of MSCs^si-TGF-β^ in inducing the transformation of LNCaP cells was examined *in vivo* and *in vitro*. As shown in Fig. 4A, MSCs^si-TGF-β^ failed to promote the growth of LNCaP cells when they were coinjected in castrated nude mice. At the same time, LNCaP cells pretreated with conditioned medium collected from MSCs^si-TGF-β^ did not exhibit a higher development speed than control group (Fig. 4B). Besides that, in androgen retreatment condition, conditioned medium obtained from MSCs^si-TGF-β^ could not lead to an androgen-independent manner in LNCaP cells (Fig. 4C,D). Taken together, the results indicated that MSCs might induce the transformation of LNCaP cells from androgen-dependent into independent manner by producing TGF-β.

### Androgen could effectively inhibite the expression of TGF-β in MSCs

**Figure 5:**
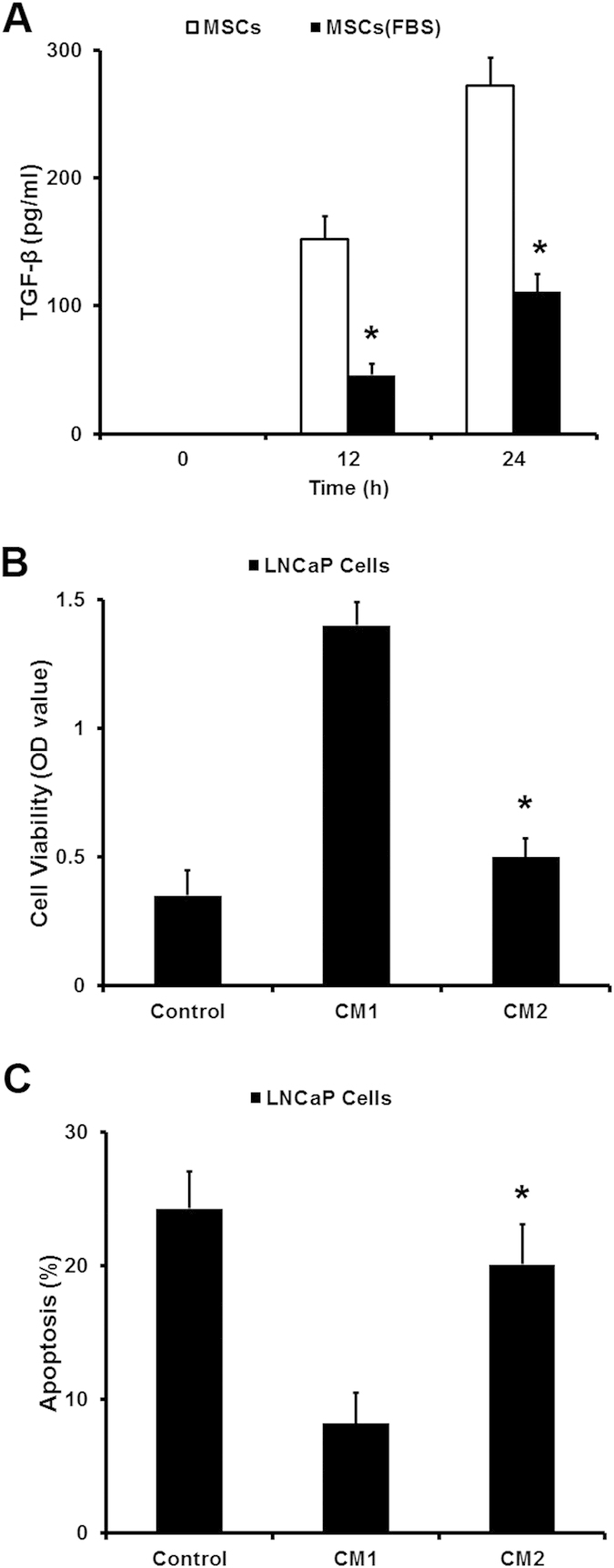
Androgen could effectively inhibited the expression of TGF-β in MSCs. (**A**) ELISA assay was employed to detect the expression of TGF-β in MSCs DMEM culture medium with or without fetal bovine serum (FBS). (**B,C**) The MSCs culture medium (DMEM with 10% FBS) treated with 10% charcoal (CM1) was used to observe their function to induce androgen-independent manner in LNCaP cells compared with the culture medium untreated with 10% charcoal (CM2). CCK8 and PI/Annexin V-FITC assay was used to examined the cell viability and apoptosis of LNCaP cells in an androgen-deprivation (**AD**) condition (*P < 0.05).

The transformation of human PCa cells from androgen-dependent into independent manner always happens during the treatment of androgen blocking therapy. So we examined the effect of androgen on the expression of TGF-β in MSCs. Interestingly, the result showed that androgen could effectively inhibit the expression of TGF-β in MSCs (Fig. 5A). As shown in Fig. 1B, when LNCaP cells were coinjected with MSCs in castrated male nude mice, MSCs demonstrated their funtion in supporting LNCaP cells growth *in vivo*. Furthermore, once we removed the FBS from the culture medium of MSCs, this medium could lead to an androgen-independent manner in LNCaP cells (Fig. 5B,C). These results illustrated that androgen blocking therapy that used in PCa treatment may induce a substantial expression of TGF-β in MSCs which leaded to the androgen-independent manner in human PCa cells.

### Correlations of MSCs with pathologic characteristics of PCa

**Figure 6:**
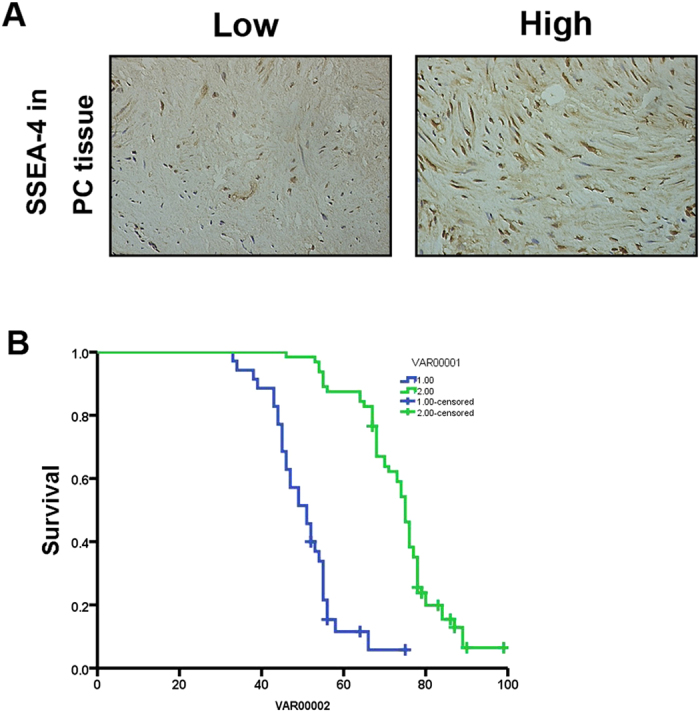
Immunohistochemical analysis of SSEA-4 expression in PCa surgical specimens. (**A**) Expression of SSEA-4 was positive in 77/99 of PCa tissues and negative in 22/99 (400 × magnification). (**B**) The patients with PCa were divided into positive- and negative-expression groups based on SSEA-4 immunostaining scores. The survival rate of the patients in the positive-expression group was significantly lower than that of patients in the negative-expression group (P = 0.033 by the log-rank test).

We also employed SSEA-4 to explore MSCs in PCa tissues by immunohistochemistry (IHC). As shown in Fig. 6A, all 99 PCa patients were divided into two groups: the high expression group (n = 77) and low expression group (n = 22). High expression of SSEA-4 was detected in 77.8% of PCa tissues (77/99). The SSEA-4 expression level was found to be significantly higher in PCa patients with pathological stage (P = 0.026), lymph node status (P = 0.012), tumour margins (P = 0.049), gleason score (P = 0.007), and biochemical recurrence (P = 0.026) (Table 1). PCa patients in high expression of SSEA-4 group had either worse OS (median survival time, 49.39 weeks versus 72.24 weeks, P = 0.033; Fig. 6B) than those with low expression of SSEA-4. The results suggested that MSCs that existed in PCa specimens might promote the development of PCa which lead to a poor prognosis of PCa patients.

## Discussion

Solid tumors are composed of tumor cells and supportive non-tumor components known as tumor stroma. There is evidence that tumor stromal cells could be derived from BM-derived progenitor cells which are believed to originate from hematopoietic stem cells as well as nonhematopoietic stem cells, such as MSCs^22^.

MSCs known as multipotent stromal precursors display inherent tropism for tumor tissue^13^. Their function in tumors has led to a great deal of interest over the past decade. It has been reported that MSCs could differentiate into various specialized cell types under certain physiological or experimental conditions^7^,^8^,^23^ and they have been recognized to contribute to the regeneration of a wide variety of organs and healing of some diseases^24^,^25^,^26^. Furthermore, MSCs also play an important role in treating various degenerative diseases and immune disorders. Therefore, MSCs have been regarded as a potential therapy for many diseases. Previous studies have shown that MSCs interact with tumor cells in a myriad of ways, which have the potential to support tumor growth, including promoting tumor vessel formation^26^, inducing immunosuppressive effect^27^, and constructing cancer stem cell niches^28^,^29^.

In order to clarify the utility of MSCs and avoid inappropriate use of MSCs in clinical therapy, we must fully understand the role of MSCs in malignant tumor, such as prostate cancer. Our results demonstrated that MSCs could affect the transformation of androgen-dependent human PCa cells into androgen-independent manner *in vivo* and *in vitro*. We know that androgen deprivation therapy is an important method for the clinical treatment of prostate cancer. However the transformation of human prostate cancer from an androgen-dependent manner to an androgen-independent manner is a lethal progression in clinical patients. It is the main obstacle that impacts the treatment during androgen deprivation therapy. Our results indicate that MSCs may support the growth of prostate cancer cells in androgen deprivation therapy, which will lead to androgen resistance in human prostate cancer cells.

As we all know, TGF-β is a key cytokine, which is related to the tumor cell invasion and migration by regulating some cytokines expression in various tumor models. We found a substantial expression of TGF-β in MSCs in androgen deprivation condition, while there was no obvious expression of IL-6 and IGF-1 in MSCs at the same condition. Blocking the effect of TGF-β by siRNA could significantly inhibit the promotive function of MSCs in PCa cells. Besides that, our results also indicated androgen might inhibit the expression of TGF-β in MSCs. On one hand, MSCs might induce the transformation of LNCaP cells from androgen-dependent into independent manner by producing TGF-β. On the other hand, androgen blocking therapy that used in PCa treatment may induce a substantial expression of TGF-β in MSCs which leaded to the androgen-independent manner in PCa cells. So androgen deprivation therapy on PCa treatment should not only focus on PCa cells. The stromal cells such as MSCs may produce an alternative signal for PCa cells to deal with survival pressure.

Furthermore, we employed SSEA-4 as a surface marker to identify MSCs in mice subcutaneous tumor models and clinical PCa tissues. The results illustrated that SSEA-4 positive MSCs were detected in PCa tissues and tumors cojected with LNCaP cells and MSCs in castrated nude mice. Furthermore,we also found that overexpression of SSEA-4 was significantly correlated with poor prognosis of PCaAnd the number of MSCs in PCa tissues was also associated with a worse overall survival (OS).

In conclusion, this study investigates the relationship among MSCs, prostate cancer cells, TGF-β and androgen. The results suggest that androgen blockade treatment in clinical PCa therapy may elicite the expression of TGF-β in MSCs, which will result in the transformation of androgen-dependent human PCa cells into androgen-independent manner. We expect that our findings will offer insights into further explore on the mechanism of prostate cancer development and provide a theoretical basis for finding new therapies for prostate cancer.

## Materials and Methods

### Cells and Animals

MSCs from the iliac crest bone marrow of multiple normal adult volunteers were isolated and cultured as described elsewhere^30^,^31^. Cells were cultured in MSCs basal medium (Invitrogen,Carlsbad, CA). To confirm stem cell isolation, all bone marrow-derived cells were tested for their multilineage capacity using standard differentiation assays^32^,^33^. Human PCa cell line LNCaP cells were purchased from the Cell Bank of Type Culture Collection of Chinese Academy of Sciences, Shanghai Institute of Cell Biology, Chinese Academy of Sciences and were cultured in RPMI1640 supplemented with 10% fetal bovine serum, streptomycin (0.1 mg/mL), penicillin (100 units/mL), and 250 nmol/L of dexamethasone at 37 °C in a 5% CO_2_ atmosphere. Nude mice, 6–8 weeks old, were purchased from Shanghai Experimental Animal Center of the Chinese Academy of Sciences, Shanghai, China. Mice in this study were housed in pathogen-free conditions. All animal experiments were approved by the guidelines of the Animal Care and Experimentation Committee of Affiliated Tumor Hospital of Guangxi Medical University, and in accordance with the guidelines of Affiliated Tumor Hospital of Guangxi Medical University.

### LNCaP murine prostate cancer model

LNCaP cells and MSCs were prepared either as single-cell type suspensions (1 × 10^6^ LNCaP cells in 200 μL PBS) or a mix of cells (1 × 10^6^ LNCaP cells and 2 × 10^5^ MSCs in 200 μL PBS). LNCaP cells (alone or mixed with MSCs) were subcutaneously administrated in the armpit area of nude mice. Mice were examined every day and tumor growth was evaluated by measuring the length and width of tumor mass. At the end of the experiment, the animals were sacrificed and tumors were removed. Tumor masses were weighed and analyzed by histology.

### Proliferation assays

Cells were plated at a density of 1 × 10^4^ cells per well in 96-well plates. After 24 h, the cancer cells cultured with medium mixed with or not 50% MSCs-conditioned medium (MSCs-CM). At 48 h, CCK8 test was performed to evaluate the extent of cell proliferation (OD values).

### Cell apoptosis assay

LNCaP cells (2 × 10^5^ cells/well) were cultured in 6-well plates to 70–80% confluence. The cells were then treated with MSCs-conditioned medium with or without androgen. PI/Annexin V-FITC assay was used to measure apoptotic cells by flow cytometry according to the manufacturer’s instruction (Keygen Biotech. Co., Ltd, Nanjing, China, Cat.KGA108). The cells were collected by trypsinization and were washed with ice cold phosphate-buffered saline (PBS). Cells were then incubated in 300 μL of 1 × binding buffer containing 5 μL Annexin V and 5 μL PI for 30 min at room temperature in the dark. Apoptosis of cells was measured on a BD FACSAriaflow cytometer (Becton Dickinson, Lincoln Park, NJ). At least 30,000 gated events were acquired from each sample. Results are expressed as the percentage of apoptotic cells (PI and Annexin V positive) in the gated cell population.

### The Conditioned Medium (CM)

MSCs were cultured with DMEM culture medium without FBS. After continuous culture for further 12 h and 24 h, the conditioned medium was collected from the supernatant media of MSCs using 0.22 μm filtration.

### Real-time PCR

The total cellular mRNA was collected with Trizol Reagent (Invitrogen). cDNA was synthesized using MMLV reverse transcriptase(Promega) and 2 μg total RNA and oligo dT18-primers. Two-microliter aliquots of cDNA were used for PCR amplification. Real-time PCR was performed in triplicate using the SYBR PrimeScript RT–PCR Kit (Takara). Primers for TGF-β mRNA determination were: 5′-GCC GAG CCC TGG ACA CCA AC-3′ (forward) and 5′-GCG CCC GGG TTA TGC TGG TT-3′ (reverse). Total sample RNA was normalized to endogenous β-actin mRNA. Thermocycler conditions included an initial hold at 50 °C for 2 minutes and then 95 °C for 10 minutes; this was followed by a two-step PCR program of 95 °C for 15 seconds and 60 °C for 60 seconds repeated for 40 cycles on an Mx4000 system (Stratagene, La Jolla, CA), on which data were collected and quantitatively analyzed. Expression level of mRNA was presented as fold change relative to an untreated control.

### Enzyme linked immunosorbent assay (ELISA)

ELISA assays were performed with a commercial ELISA kit (R&D Systems, Minneapolis, MN). The MSCs conditioned medium was collected for further experiment. Assays were performed in duplicate, and readings were compared with standard curves obtained with standard protein provided with the kit. Means and standard deviations of concentrations in triplicate samples were compared by t-test.

### Short interfering RNA (siRNA) synthesis and transient transfection

Three siRNA sequences of TGF-β were designed using Oligoengine software and verified by nucleotide BLAST searches. Three candidate sequences and a control sequence with no significant homology were listed in Table 2. Cells (1–3 × 10^6^) growing to 50–60% confluence in 10 cm petri dishes were transfected with siRNA sequences or their corresponding mock sequences using a Lipofectamine 2000 kit (Invitrogen, Cat.11668-019) with the procedure provided by the manufacturer. Cells were observed under a fluorescence microscope and harvested 48 h after transfection.

### Immunohistochemistry staining

Tumor tissue sections (4 μm thick) were cut and mounted on 3-aminopropyl-triethoxy-silane-coated slides. Incubation with monoclonal anti-human SSEA-4 antibody (eBioscience) was performed at room temperature for 1 h, after blocking endogenous peroxidase activity. Detection of the primary antibody was performed using rabbit anti-mouse antibody (DAKO A/S, Denmark) and streptavidin–biotin–horseradish peroxidase complex (SABC/HRP, DAKO A/S, Denmark). The peroxidase reaction was visualized using diaminobenzidine/H2O2 [0.05% (w/v)/0.03% (v/v)].

### Patients and Tissue Specimens

Specimens of PCa tissues were obtained from 99 PCa patients who underwent prostate resection at Affiliated Tumor Hospital of Guangxi Medical University from February 2009 to December 2013. These patients included a median age of 61 years (range: 55–72), and all of the specimens were subjected to immunohistochemisty (IHC). Informed written consent was obtained from all patients studied and the study protocol was approved by the Ethics Committee of Affiliated Tumor Hospital of Guangxi Medical University.

### Statistical analysis

Statistical analysis of the data was done by using GraphPad Prism 5. Student’s t-test was used to compare between mean values of two groups. Final values are expressed as mean S.E.M. A difference of at least p < 0.05 was considered statistically significant.

## Additional Information

**How to cite this article**: Cheng, J. *et al.* The role of mesenchymal stem cells in promoting the transformation of androgen-dependent human prostate cancer cells into androgen-independent manner. *Sci. Rep.* **6**, 16993; doi: 10.1038/srep16993 (2016).

## Figures and Tables

**Table 1:** Relationship between SSEA-4 expression and the clinicopathological features of 99 patients with prostate cancer tissue.

Factor	Category	Total no. of patients	SSEA-4 negative (n = 22)	SSEA-4 positive (n = 77)	*P*
Age (years)	>65	43	9(40.9)	34(44.1)	0.786
	≤65	56	13(59.1)	43(55.9)	
Pathological stage (pT)	pT2 – pT3a	43	5(22.7)	38(49.4)	0.026*
	pT3b	56	17(77.3)	39(50.6)	
Lymph node status	Positive	58	18(81.8)	40(51.9)	0.012*
	Negative	41	4(18.2)	37(48.1)	
Tumour margins	Positive	21	8(36.6)	13(16.9)	0.049*
	Negative	78	14(63.4)	64(83.1)	
Capsular invasion	No invasion	66	12(54.5)	54(70.1)	0.171
	Invasion	33	10(45.5)	23(29.9)	
PSA concentration (ng/mL) before surgery	Median (range)		17.40(1.22–105)	21.59(3.38–91.45)	<0.001**
	Mean	21.22	25.94		
Gleason score	Gleason≤6	27	11(50.0)	16 (20.8)	0.007*
	Gleason≥7	72	11(50.0)	61(79.2)	
Biochemical recurrence	Yes (≥0.2 ng/mL)	76	13(59.1)	63(81.8)	0.026*
	No (<0.2 ng/mL)	23	9(40.9)	14(18.2)	

**Table 2:** Sequence of the oligonucleotides for siRNA construct-making assays.

Assays	Gene	Sequence (5′→3′)	
TGFβ1 siRNA	Sequence 1	Sense	CACUGCAAGUGGACAUCAATT
		Antisense	UUGAUGUCCACUUGCAGUGTT
	Sequence 2	Sense	GCAAGACUAUCGACAUGGATT
		Antisense	UCCAUGUCGAUAGUCUUGCTT
	Sequence 3	Sense	GCAUAUAUAUGUUCUUCAATT
		Antisense	UUGAAGAACAUAUAUAUGCTT
	Control	Sense	UUCUCCGAACGUGUCACGUTT
		Antisense	ACGUGACACGUUCGGAGAATT
